# Effect of Recombinant Human Epidermal Growth Factor Associated with Conventional Drug Therapy on the Dry Eye Symptom Score in Patients with Dry Eyes after Cataract Surgery: A Systematic Review and Meta-Analysis

**DOI:** 10.1155/2022/5142851

**Published:** 2022-09-30

**Authors:** Changce Sun, Xiaojun Zhang

**Affiliations:** Department of Ophtalmology, The Second Affiliated Hospital of Nanjing Medical University, Nanjing, China

## Abstract

**Objective:**

This study aimed to systematically assess the effect of recombinant human epidermal growth factor (rhEGF) associated with conventional drugs on the score of dry eye symptoms in patients with dry eyes after cataract surgery.

**Methods:**

The online database was searched for the clinical controlled trials of rhEGF associated with conventional drugs in the therapy of dry eyes after cataract surgery. Until now, the retrieval timeframe is based on the establishment of the database. Separately, two researchers extracted the data. The bias risk of each included literature was assessed.

**Results:**

Eight clinical controlled studies were finally included, with 878 samples. The success rate of the study group was greatly higher, and the difference was statistically significant (*P* < 0.05). The fluorescein staining (FL) score of the research group after treatment was lower, and the difference was statistically significant (*P* < 0.05). Compared with the control group, the tear break up time (BUT) of the study group after treatment was notably prolonged. The dry eye symptom score of the research group after treatment was notably lower, and the difference was statistically significant (*P* < 0.05).

**Conclusion:**

Sodium hyaluronate associated with rhEGF eye drops is successful to treat xerophthalmia after cataract operation. It successfully promotes corneal healing, promotes tear film stability, and increases basic tear secretion. This treatment scheme is worth popularizing and applying in clinics.

## 1. Introduction

The disease in which the lens becomes cloudy due to degeneration and degeneration of the lens due to the combined action of various factors is called cataract. With the acceleration of the aging process of China's population, the incidence of cataract is also gradually increasing. Epidemiological surveys have found that in male patients, the incidence of cataracts in the age group of 45–49 years was 3.23%, while that in the age group of 85–89 years was 65.78%. The same trend was also observed in female patients [1]. Cataracts have become one of the most common diseases causing blindness in the world due to their obvious impact on vision [2]. When the visual acuity of cataract patients is notably reduced, the quality of life of patients will also decline. Therefore, treating cataracts to restore sight to patients is the common responsibility and goal of ophthalmic medical staff.

Nowadays, surgery is the most successful way to notably improve the vision of cataract patients [3]. Patients with cataracts are generally older, who have many systemic diseases in the aging of the body, making the postoperative healing time longer than that of young people. Therefore, surgical approaches that can cause minimal damage to the incisional tissue, incisions that do not require sutures, shorter operative durations, and significantly improved postoperative vision are recommended. Ultrasound emulsification with intraocular lens implantation has become the most commonly used surgical method because of its short operation time, low surgical requirements, and mature technology [4].

Sodium hyaluronate can absorb a large number of negatively charged anions with enough water, thus lubricating the eye surface. At the same time, sodium hyaluronate has the property of mucin so that tears can have high viscosity and stay on the eye surface for a longer time to form a good protective film; through close binding of fibrin, the connection and extension of the corneal epithelium can be promoted, and corneal epithelial regeneration can be accelerated [5]. Xerophthalmia after cataract surgery is a common clinical inflammatory disease of the eye surface, which can cause abnormal tear quality. The incidence of xerophthalmia in China is as high as 30% [6]. Dry eye can cause severe discomfort, causing instability of the tear film, leading to problems with the surface tissue of the dry eye and great damage to visual function [7]. Therefore, it is necessary to carry out targeted treatment to improve the quality of life and avoid affecting visual function.

There are many kinds of artificial tears, among which sodium hyaluronate is the most used because of its good viscosity and ductility [8]. The anti-inflammatory effect of sodium hyaluronate can also relieve local irritation on the ocular surface [9, 10]. rhEGF has been shown to enhance the quantity of convoluted goblet cells in patients suffering from dry eye, which successfully facilitates the growth and differentiation of tear film and epithelial structures of the cornea, reducing corneal epithelial damage, promoting changes in tear film quality, and alleviating dry eye signs [11]. Previous studies have found that patients who undergo phacoemulsification have an obvious tendency to dry eyes (DED) in the short term after phacoemulsification. Recombinant bovine basic fibroblast growth factor (rbFGF) can enhance basal tear secretion, subjective dry eye sensation score and corneal fluorescent staining score in patients with xerophthalmia for one month [12, 13].

Many clinical data have confirmed that the combination of sodium hyaluronate without preservatives and rhEGF is potent after cataract surgery, but there are great differences between different research designs and a variety of evaluation indicators. Only the success of the research design or the improvement of evaluation indicators can prove the clinical value of recombinant human epidermal growth factor combined with sodium hyaluronate after cataract surgery, and the results are not convincing. It still needs to be supported by high-quality research evidence. Under this background, further related research is very necessary. More authoritative scientific research is needed to demonstrate the effect of rhEGF associated with sodium hyaluronate when treating dry eyes after cataract surgery, in order to offer a theoretical basis for the promotion and application of this treatment. Therefore, this study made a systematic, quantitative, and comprehensive analysis of the results of similar independent studies through meta-analysis to assess the promoting effect of rhEGF associated with sodium hyaluronate.

## 2. Materials and Methods

### 2.1. Sources and Retrieval Methods of Documents

PubMed, EMBASE, Science Direct, Cochrane Library, China National Knowledge Infrastructure (CNKI), VIP Full-Text Database (VIP), Wanfang Database, and Chinese Biomedical Literature Database (CBM) were searched. A literature review was used to collect relevant data about rhEGF associated with conventional drugs after cataract surgery. A literature search was conducted with free words + subject words, with the key words of recombinant human epidermal growth factor; rhEGF; combined/combination therapy; cataract surgery; dry eye symptoms; xerophthalmia; improvement; meta-analysis from January 2010 to April 2022.

### 2.2. Literature Inclusion and Exclusion Criteria

#### 2.2.1. Literature Inclusion Criteria

*(1) Research types*. All the clinical controlled trials of rhEGF and conventional drugs after cataract surgery.*(2) Participants.* Patients with dry eyes after cataract surgery and those who met the diagnostic criteria were included in this study [14].*(3) Intervention.* The control group was cured with sodium hyaluronate eye drops, and the study group was cured with rhEGF associated with sodium hyaluronate eye drops.

#### 2.2.2. Literature Exclusion Standard

(1) It was not a case-control study; (2) it was not possible to use the data since the report was incomplete; (3) duplication of studies, taking the latest research; (4) the related literature was reviewed; (5) clinical cases.

### 2.3. Quality Evaluation and Data Extraction

1. A bias risk assessment was included in the study.
2. Literature screening and data extraction: the contents of data extraction were as follows: (1) basic information: author, publication time, and the number of cases; (2) intervention: scheme and course of treatment; (3) outcome index: corneal fluorescein staining (FL) score, tear break up time (BUT), tear secretion test (SITT), overall successful rate, and dry eye symptom score.

### 2.4. Statistical Processing

According to the previous study [15], the standardized mean difference (SMD) with Hedges' *g* was chosen as the measure of the effect. The effect size was calculated using a random-effect model with a restricted maximum-likelihood (REML) and considered a large, moderate, and small effect with respect to the SMD values of 0.8, 0.5, and 0.2, respectively. The heterogeneity among the studies included in a meta-analysis was assessed using Cochrane's *Q*, tau-squared, and I-squared (I^2^). Cochrane's *Q* test quantifies the total variance and generates a *p* value that determines that the heterogeneity is present. Tau-squared indicates the true variance that is the between-study variance, while I^2^ represents the percentage of the total variance that is due to the true variance. The degree of heterogeneity is said to be low, moderate, and high, with I^2^ values of 25%, 50%, and 75%. RevMan 5.3 software was adopted for meta-analysis. HR and its 95% CI were employed as effect analysis statistics for OS and PFS, and the risk ratio and 95% CI were employed as effect analysis statistics for binary variables. P and I^2^ values in heterogeneity test results were adopted to determine whether there was statistical heterogeneity among the results. *P* > 0.10, I^2^<50% indicated that there was no statistical heterogeneity among the research results, and a fixed-effect model was used for combined analysis. *P* ≤ 0.10, I^2^ ≥ 50% indicated statistical heterogeneity among the research results, and a random-effect model was adopted for combined analysis. The test level of the meta-analysis was set as *α* = 0.05. Eggers' test was used to examine the funnel plot asymmetry. Whenever this test was significant with a *p* value of less than 0.1, we used the trim and fill method to correct the funnel plot and adjust the effect size for potential publication bias.

## 3. Results and Analysis

### 3.1. Results of Literature Retrieval and the Basic Situation of Literature Inclusion

**Figure 1:**
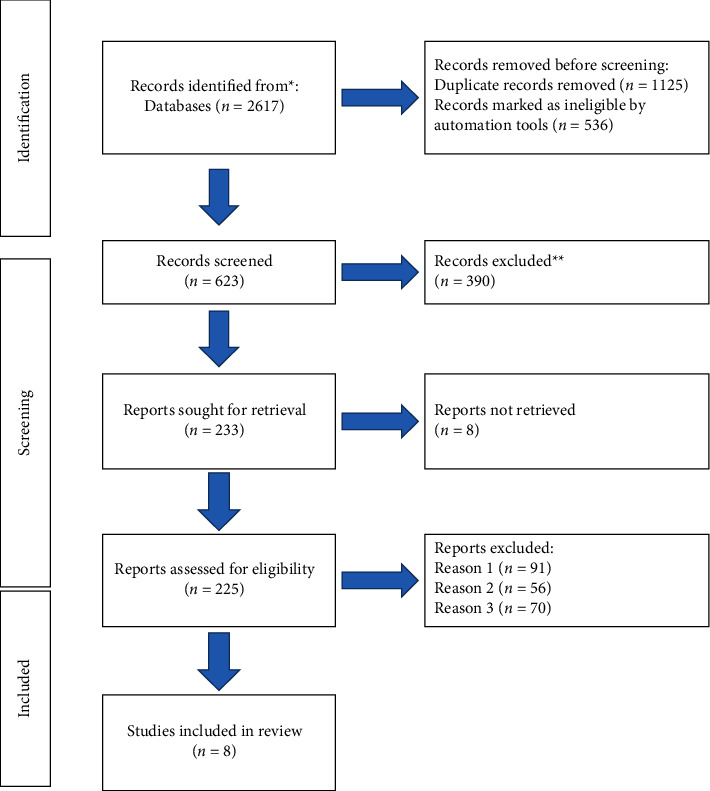
Illustration of literature screening.

Eight clinical controlled studies were finally included in this study [16–23]. A total of 878 samples were assessed by meta-analysis. The illustration of the literature screening method is shown in Figure 1. The basic characteristics of literature reviews included in our research are shown in Table 1.

### 3.2. Evaluation of the Quality of the Methodology Included in the Literature

**Figure 2:**
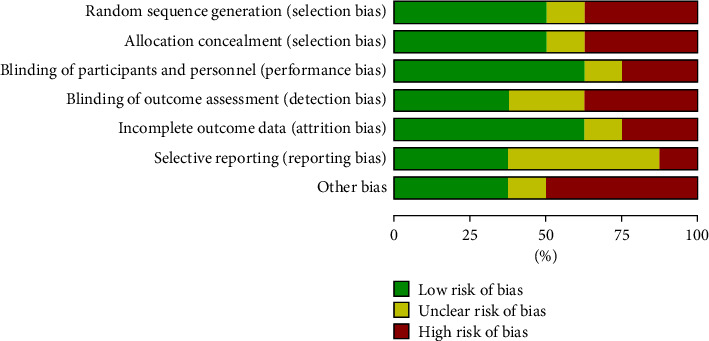
Risk bias chart.

**Figure 3:**
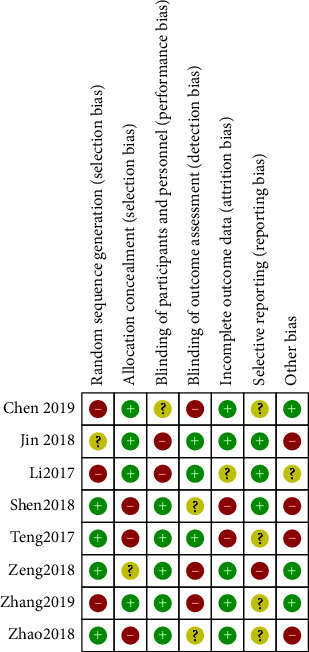
Summary chart of risk bias.

Only 2 literature reviews explained the specific random method, so the risk was uncertain. All the literature did not mention whether the allocation was hidden or not, so it was defined as risk uncertainty. The blind method was not mentioned in all the literature, so the performance risk was defined as a high risk, and the detection risk was defined as a low risk. All literature reported complete data and no missing case data, which was a low risk. All literature did not obtain their trial plans, so they were classified as risk uncertainty. No other risks were found in all literature reports, so they were classified as low risk (Figures 2 and 3).

### 3.3. Meta-Analysis Result

#### 3.3.1. Treatment Successful Rate

**Figure 4:**
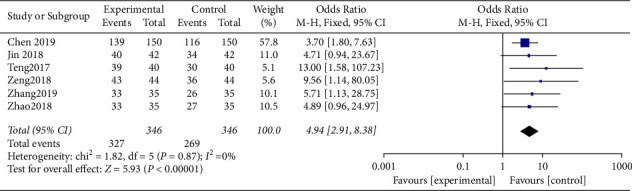
Forest analysis map of the comparison of treatment efficiency.

The heterogeneity test was chi^2^ = 1.82, d*f* = 5, *P*=0.87, I^2^ = 0%, without obvious heterogeneity. According to the analysis of the fixed-effect model (Figure 4), the success rate of the study group was notably higher, and the difference was statistically significant (*P* < 0.05).

#### 3.3.2. Corneal Fluorescein Staining (FL) Score

**Figure 5:**
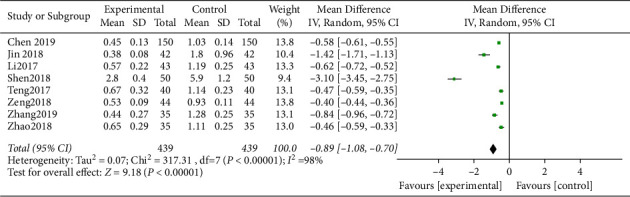
FL score after treatment.

FL scores of both cohorts was displayed chi^2^ = 317.31, d*f* = 7, *P* < 0.00001, I^2^ = 98%, with obvious heterogeneity (Figure 5). The random-effect model analysis suggested that the FL score of the study group was notably lower, and the difference was statistically significant (*P* < 0.05).

#### 3.3.3. Tear Film Rupture Time (BUT)

**Figure 6:**
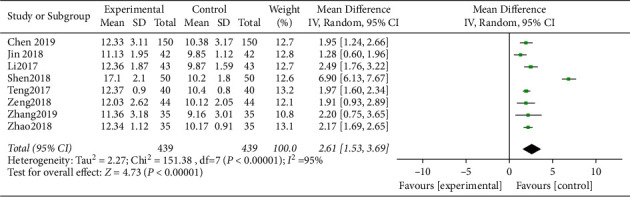
Forest analysis map of comparison of BUT between the two groups after treatment.

BUT status of heterogeneity data was Chi^2^ = 151.38, d*f* = 7, *P* < 0.00001, I^2^ = 95% (Figure 6). It indicated that there was obvious heterogeneity among the included research data. Compared with the control group, BUT of the study group after treatment was largely prolonged, and the difference was statistically significant (*P* < 0.05).

#### 3.3.4. Schirmer Test (SIT)

**Figure 7:**
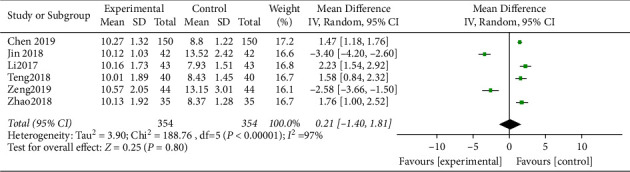
Forest analysis map of the condition of slab after treatment.

SIT in heterogeneity exhibited Chi^2^ = 188.76, d*f* = 5, *P* < 0.00001, I^2^ = 97%. The random-effect model analysis (Figure 7) indicated that no obvious differences were discovered in SIT.

#### 3.3.5. Dry Eye Symptom Score

**Figure 8:**
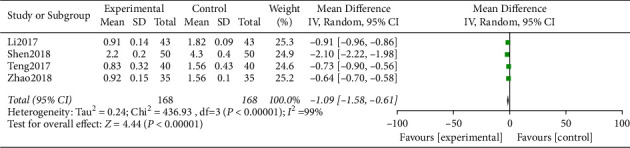
Forest analysis map of the dry eye symptom score.

The scores of the heterogeneity data were Chi^2^ = 436.93, df = 3, *P* < 0.00001, I^2^ = 99%. The random-effect model analysis showed that (Figure 8) the dry eye symptom score of the study group after treatment was notably lower, and the difference was statistically significant (*P* < 0.05).

#### 3.3.6. Publication Bias Analysis

**Figure 9:**
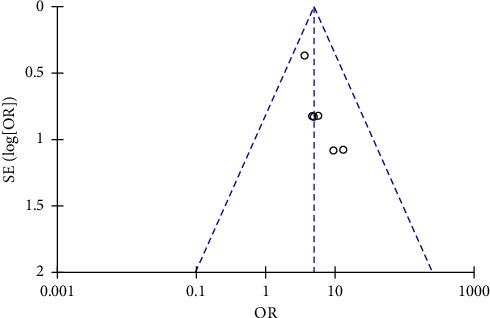
Funnel chart with successful treatment.

The results suggested that there was a certain publication bias in the included literature (Figure 9). This may be relevant to the heterogeneity of the study and the small number of included literature reports.

## 4. Discussion

This study made a systematic, quantitative, and comprehensive analysis of the results of similar independent studies through meta-analysis to assess the promoting effect of rhEGF associated with sodium hyaluronate. As China enters an aging society, the incidence of cataract, a common disease in the elderly, is increasing year by year. Once the disease occurs, it will have a great adverse impact on work and life. Surgical treatment for cataract can achieve satisfactory results. However, cataract surgery will cause partial damage to the tear secretion function and cornea. After surgical treatment, surgical trauma will lead to inflammatory reaction, which will lead to a notable increase in the probability of dry eye [24]. The tear film is directly related to the body's vision, and once dry eye occurs, it can cause great damage to the stability and integrity of the tear film, making it difficult for the tear film to attach successfully. The disease will also lead to an increase in tear osmolarity, which can easily lead to corneal perforation. As the disease progresses, it can also lead to the serious consequence of blindness [25]. The treatment success rate of the study group was greatly higher, and the dry eye symptom scores of the study group were lower. Conventional drugs and rhEGF could notably improve the therapeutic effect, which notably relieve dry eye symptoms, being consistent with the conclusions of previous studies.

The main component of sodium hyaluronate eye drops is sodium hyaluronate, which is a mucopolysaccharide [9]. It binds to fibronectin to promote epithelial cell attachment and extension. In addition, its molecules retain many water molecules and are biocompatible and nonliquid, providing excellent water retention for lubrication and moisture. It can not only promote the healing of corneal epithelial cell damage but also reduce dryness and viscosity and reduce discomfort when blinking. At the same time, sodium hyaluronate has certain plasticity, no antigenicity, and no immune response of the body occurs.

A variety of components of human tissues, organs, and body fluids play a positive role in the repair of corneal epithelial injury [26]. Sodium hyaluronate eye drops and rhEGF can play a synergistic role in maintaining the stability of the tear film. The results of Cheng showed that the combination could notably improve the symptoms of xerophthalmia patients by delaying the rupture of the tear film [27]. The results of Kang and Wan showed that the combination of polyethylene glycol eye drops can achieve a good clinical effect when treating dry eyes [28].

BUT is one of the important indicators to evaluate tear film stability [29]. SIT is used to evaluate the amounts of tears in the conjunctival sac. FL is used to evaluate the degree of ocular surface damage, which is an indirect indicator to evaluate tear film stability. Meta-analysis of the FL scores, BUT, and SIT was carried out. The FL score of the study group was notably lower. BUT in the study group after treatment was notably longer; however, no notable difference was exhibited in SIT after treatment. It is suggested that rhEGF associated with diclofenac sodium can enhance the lacrimal gland function of patients with xerophthalmia after cataract surgery. First, sodium hyaluronate has good biocompatibility and can combine with fibrin to enhance the water retention capacity of the tear film and greatly improve the moisturizing ability of the cornea. Conventional drugs (sodium hyaluronate eye drops) associated with rhEGF can play a synergistic effect, and the two drugs can achieve complementary advantages, maximize the clinical efficacy, and improve patients' clinical symptoms and physiological indicators. The application of rhEGF is helpful to repair corneal epithelial injury, maintain normal corneal epithelial results, change the quality of the tear film, and alleviate the symptoms of patients. The viscosity of the drug successfully improves the problem that the eyelids do not blink easily, making it an excellent carrier for eye drops. In addition, rhEGF can promote the regeneration of corneal collagen synthesis, repair the corneal epithelium, stroma, and endothelium, stimulate the proliferation of nerve fiber cells, repair the peripheral nerve fibers injured during operation, and restore the transport of acetylcholine and cholinesterase, which can help restore the function of the lacrimal gland. Furthermore, rhEGF also contains glycerol and mannitol, which can moisturize the eye and cornea and restore the normal physiology of the cornea and tear glands, stabilizing the tear film. The limitations of the study are as follows: (1) the inclusion and exclusion criteria are relatively strict, and the final number of included literature reports is relatively small; (2) the source of heterogeneity cannot be found through subgroup analysis, which needs to be followed up by scholars, and the results of this study need to provide more support. More high-quality randomized controlled trials are needed to verify.

## 5. Conclusion

To sum up, rhEGF associated with conventional drugs is of high value when treating dry eyes after cataract surgery, which can successfully relieve the symptoms of dry eye, and enhance the corneal repair ability and BUT, which is suitable for clinical application. It is worth popularizing in clinical practice.

## Figures and Tables

**Table 1:** Basic characteristics of literature.

Literature included	Year of publication	N (C/T)	Intervention method		Outcome index	Treatment time	Grouping method	Blind or not
			C	T				
Zeng [16]	2018	44/44	Sodium hyaluronate eye drops	Sodium hyaluronate eye drops + rhEGF	①②③④	4 weeks	Random, but the method is not specified	No
Chen [17]	2019	150/150	Sodium hyaluronate eye drops	Sodium hyaluronate eye drops + rhEGF	①②③④	4 weeks	Random, but the method is not specified	No
Jin [18]	2018	42/42	Sodium hyaluronate eye drops	Sodium hyaluronate eye drops + rhEGF	①②③④	2 months	Random, but the method is not specified	No
Li [19]	2017	43/43	Sodium hyaluronate eye drops	Sodium hyaluronate eye drops + rhEGF	①②③⑤	4 weeks	Random number method	No
Shen [20]	2018	50/50	Sodium hyaluronate eye drops	Sodium hyaluronate eye drops + rhEGF	①②⑤	4 weeks	Order of admission	No
Teng [21]	2017	40/40	Sodium hyaluronate eye drops	Sodium hyaluronate eye drops + rhEGF	①②③④⑤	4 weeks	Random, but the method is not specified	No
Zhang [22]	2019	35/35	Sodium hyaluronate eye drops	Sodium hyaluronate eye drops + rhEGF	①②④	8 weeks	The method is not specified	No
Zhao [23]	2018	35/35	Sodium hyaluronate eye drops	Sodium hyaluronate eye drops + rhEGF	①②③④⑤	4 weeks	Random number table method	No

*Note.* C: control group; T: study group; rhEGF: rhEGF; ①: treatment successful rate; ②: FL Scoring; ③: BUT; ④: SIT; ⑤: dry eye symptom score.

## Data Availability

The datasets used and analyzed during the current study are available from the corresponding author upon reasonable request.
